# Supplementary figures and images for: Identification of miR-194-5p as a potential biomarker for postmenopausal osteoporosis
Source: PeerJ. 2015 May 21;3:e971. doi: 10.7717/peerj.971 (PMC4451039; doi:10.7717/peerj.971)

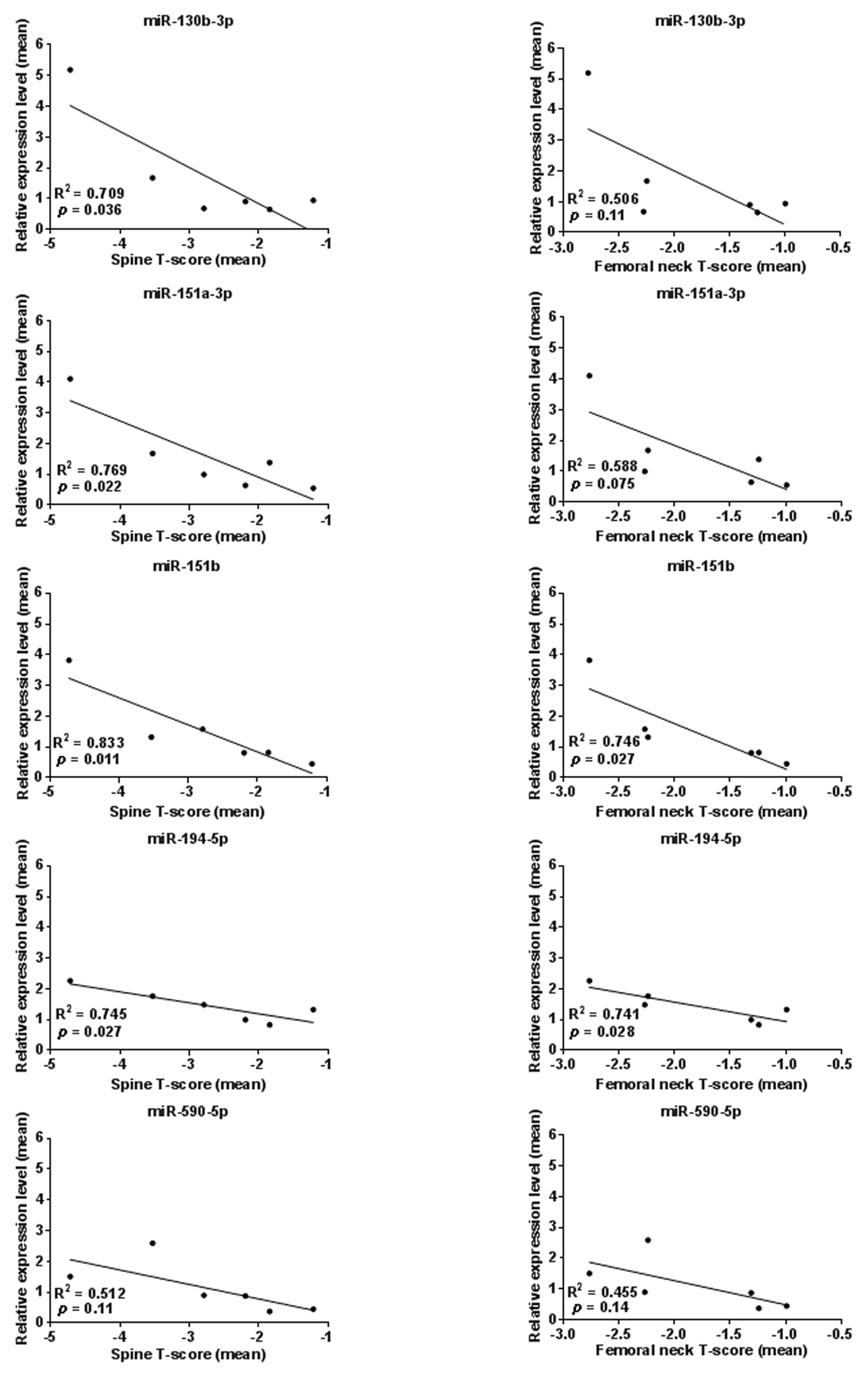

Supplement: Figure S1 — Correlation between mean miRNA expression and mean T-score (spine T-score or femoral neck T-score, n = 6). Mean miRNA expression is the mean measured value (n = 4) of each pooled RNA sample by qRT-PCR; mean T-score is the average T-score of the participants in the corresponding subgroup. [file peerj-03-971-s001.png]

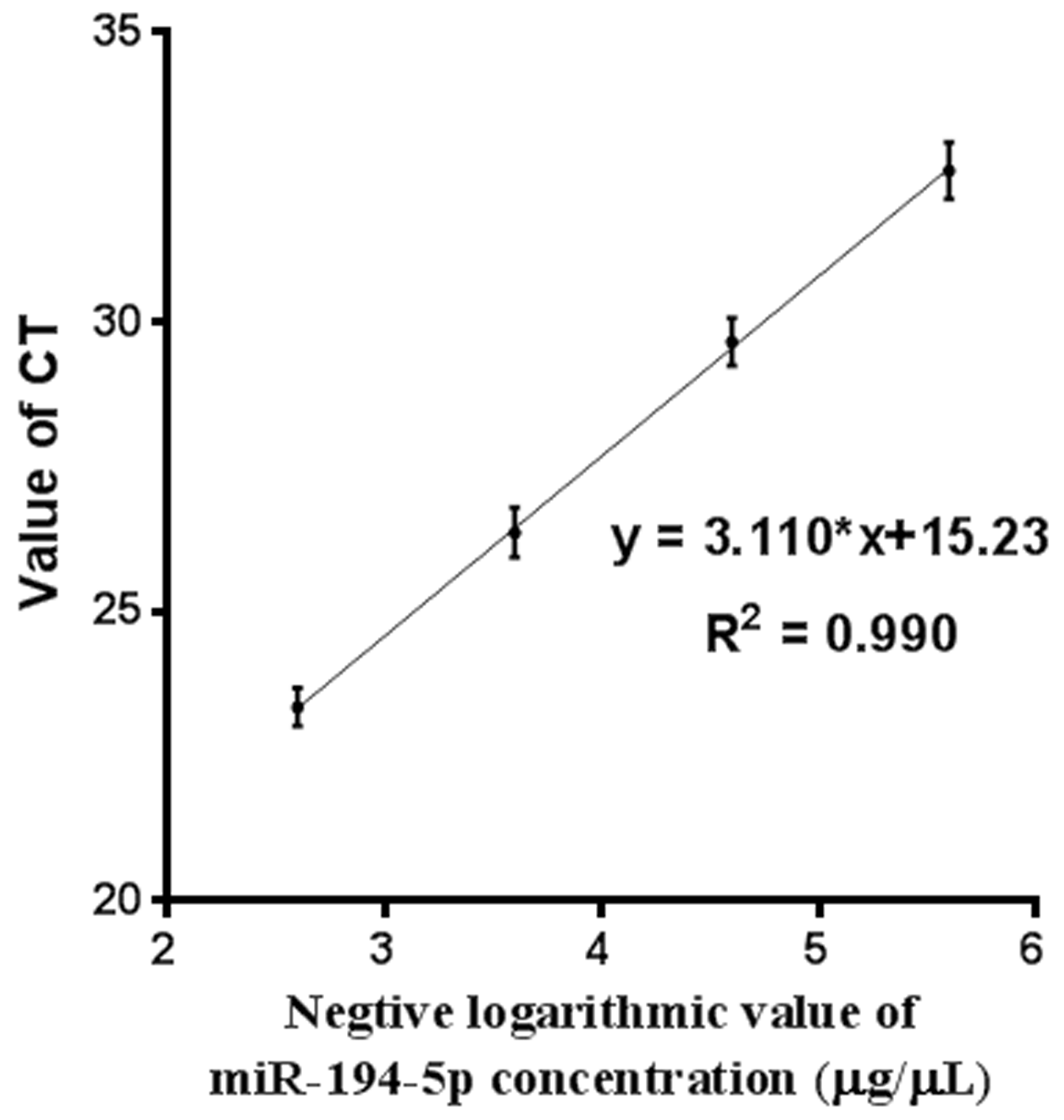

Supplement: Figure S2 — Evaluation method for miR-194-5p detection using qRT-PCR. A linear relationship was observed between the negative logarithmic value of the concentration of synthetic miR-194-5p and the CT value detected by qRT-PCR (R2 = 0.990). This experiment was repeated three times. [file peerj-03-971-s002.png]
