# Supplementary material for: Identification of miR-194-5p as a potential biomarker for postmenopausal osteoporosis
Source: PeerJ. 2015 May 21;3:e971. doi: 10.7717/peerj.971 (PMC4451039; doi:10.7717/peerj.971)
Supplement: Table S2 — Primer sequences of the genes involved in this study (human). [file peerj-03-971-s004.docx]

**Table S2.** Primer sequences of the genes involved in this study (human).

| Gene | Forward primer (5'-3') | Reverse primer (5'-3') |
| --- | --- | --- |
| miR-130b-3p | GCGGCGGCAGTGCAATGATGAAAG | ATCCAGTGCAGGGTCCGAGG |
| miR-151a-3p | GGGCTAGACTGAAGCTCC | CAGTGCGTGTCGTGGAGT |
| miR-151b | GGGTCGAGGAGCTCAC | CAGTGCGTGTCGTGGAGT |
| miR-194-5p | GCGGCGGTGTAACAGCAACTCC | ATCCAGTGCAGGGTCCGAGG |
| miR-590-5p | GCGGCGGGAGCTTATTCATAAAAG | ATCCAGTGCAGGGTCCGAGG |
| miR-660-5p | GCGGCGGTACCCATTGCATATC | ATCCAGTGCAGGGTCCGAGG |
| U6 | GCTTCGGCACATATACTAAAAT | CGCTTCACGAATTTGCGTGTCAT |
